# Methylotrophic bacteria with cobalamin-dependent mutases in primary metabolism as potential strains for vitamin B_12_ production

**DOI:** 10.1007/s10482-022-01795-9

**Published:** 2022-11-17

**Authors:** Darya Dudko, Dirk Holtmann, Markus Buchhaupt

**Affiliations:** 1 Microbial Biotechnology, DECHEMA Research Institute, Theodor-Heuss-Allee 25, 60486 Frankfurt am Main, Germany; 2 Faculty Biology and Chemistry, Justus-Liebig-Universität Gießen, Ludwigstraße 23, 35390 Gießen, Germany; 3 Institute of Bioprocess Engineering and Pharmaceutical Technology, University of Applied Sciences Mittelhessen, Wiesenstr. 14, 35390 Gießen, Germany

**Keywords:** Vitamin B_12_, Pseudovitamin B_12_, Cobalamin, LC–MS, Methylotroph, Ethylmalonyl-CoA pathway

## Abstract

Several bacterial species are known for their ability to synthesize vitamin B_12_ but biotechnological vitamin B_12_ production today is restricted to *Pseudomonas denitrificans* and *Propionibacterium freudenreichii*. Nevertheless, the rising popularity of veganism leads to a growing demand for vitamin B_12_ and thereby interest in alternative strains which can be used as efficient vitamin B_12_ sources. In this work, we demonstrate that methylotrophic microorganisms which utilize the ethylmalonyl-CoA pathway containing B_12_-dependent enzymes are capable of active vitamin B_12_ production. Several bacteria with an essential function of the pathway were tested for vitamin B_12_ synthesis. Among the identified strains, *Hyphomicrobium* sp. DSM3646 demonstrated the highest vitamin B_12_ levels reaching up to 17.9 ± 5.05 µg per g dry cell weight. These relatively high vitamin B_12_ concentrations achieved in simple cultivation experiments were performed in a mineral methanol medium, which makes *Hyphomicrobium* sp. DSM3646 a new promising cobalamin-producing strain.

**Supplementary Information:**

The online version contains supplementary material available at 10.1007/s10482-022-01795-9.

## Introduction

Vitamin B_12_ (cobalamin) is a water-soluble vitamin which plays a very important role in various metabolic processes in human body. The recommended daily dose for adults is 2.4 µg per day (Institute of Medicine 1998) and the inadequate intake is one of the reasons for vitamin B_12_ deficiency (Green et al. 2017). Several groups are at a particular risk of vitamin B_12_ deficiency. Previous studies have reported the prevalence of the deficiency in the risk groups reaching over 20% among elderly people (Andrès et al. 2004), 25% in pregnant women (Sukumar et al. 2016) and up to 86.5% in adult vegans (Pawlak et al. 2014). The cobalamin deficit in these groups is caused by different reasons but they all are susceptible to symptoms of vitamin B_12_ deficiency which include megaloblastic anemia, leukopenia, thrombocytosis and many other clinical manifestations (Langan and Goodbred 2017).

The importance of vitamin B_12_ for humans has become clear in the 1920s after two American physicians, Minot and Murphy, demonstrated the role of their so-called “extrinsic factor” in the treatment of pernicious anemia (Martens et al. 2002). After years of further research, the complete chemical synthesis of cobalamin was achieved by Woodward and Eschenmoser in 1972 (Eschenmoser and Wintner 1977). Nevertheless, the described synthesis procedure included about 70 steps, which makes it economically disadvantageous (Martens et al. 2002). Therefore, microbial synthesis is used as the exclusive strategy for industrial vitamin B_12_ production nowadays.

Although there are several cobalamin-producing species (Perlman 1959), only few of them are applied for the large scale industrial production of vitamin B_12_ (Fang et al. 2017). *Propionibacterium freudenreichii* is well known for its ability to synthesize the active form of vitamin B_12_ (Deptula et al. 2015). Due to this feature, *P. freudenreichii* has already been investigated for its ability to fortify various food sources in several studies (Chamlagain et al. 2017; van Wyk et al. 2011). Other investigations described different *Lactobacillus reuteri* strains, which can be applied for the B_12_ fortification of plant-based foods (Molina et al. 2012; Gu et al. 2015). However, *Lactobacillus* species were shown to produce a vitamin B_12_ analogue called pseudovitamin B_12_ in other studies (Crofts et al. 2013; Santos et al. 2007). The lower ligand of the active form of vitamin B_12_ is represented by 5,6- dimethylbenzimidazole (DMBI), while pseudovitamin B_12_ carries adenine as the lower ligand in its structure and is inactive in humans (Watanabe et al. 2013). For this reason, identification of new bacterial strains, which are able to produce the active vitamin B_12_ and, therefore, can be useful for food fortification, is of great importance.

Methylotrophs are a versatile group of bacteria associated with plants. Reduced C1-compounds can be used by methylotrophs as energy and carbon source for the growth and respective species were found to produce phytohormones and other plant growth-stimulating factors (Ivanova et al. 2006; Trotsenko et al. 2001). One group of methylotrophs assimilates C1 compounds via the serine cycle elucidated by Quayle and coworkers (Large et al. 1961, 1962a, b; Large and Quayle 1963) which requires continuous regeneration of glyoxylate. This occurs in many organisms via the widespread glyoxylate cycle, while some serine cycle-methylotrophs lack the isocitrate lyase, an essential enzyme of the glyoxylate pathway (Anthony 1982). It was proposed that methylotrophs operate an alternative pathway for glyoxylate regeneration and after more than 30 years of investigation the ethylmalonyl-CoA pathway (EMCP) could be identified (Erb et al. 2007), while the activity of EMCP upon C1 assimilation in *M.extorquens* was demonstrated by Smejkalova et al. 2010. This pathway includes two vitamin B_12_-dependent enzymes, the ethylmalonyl-CoA mutase and methylmalonyl-CoA mutase (Erb et al. 2008; Smejkalova et al. 2010). In this context, there have been reports providing evidence for cobalamin production in methylotrophic bacteria, especially in the genus *Methylobacterium* (Danilova et al. 2004; Trotsenko et al. 2001; Ivanova et al. 2006).

The aim of this work was to identify microbial species with high vitamin B_12_ production capabilities due to an essential function of the compound in their primary metabolism. Since *Methylorubrum extorquens* (formerly *Methylobacterium extorquens*) is able to grow on methanol as sole C-source without vitamin B_12_ supplementation, we proposed that this organism is capable of cobalamin production. Moreover, we searched for additional microorganisms having the key enzymes of the ethylmalonyl-CoA pathway and analyzed them for the production of the active form of vitamin B_12_.

## Materials and methods

### Identification of strains with probably functional EMCP

To identify microorganisms that possess a functional EMCP, we analyzed bacterial genome sequences for the presence of a crotonyl-CoA carboxylase/reductase (Ccr)-encoding gene. To exclude strains, that additionally contain the glyoxylate pathway, we considered only bacteria containing no isocitrate-lyase (Icl)-encoding gene. The genome dataset used as starting point was created in house from all bacterial genomes available at the Microbial Nucleotide Blast homepage (http://blast.ncbi.nlm.nih.gov/Blast.cgi?PAGE_TYPE=BlastSearch&BLAST_SPEC=MicrobialGenomes). If sequences similar to the *M. extorquens* AM1 Ccr protein sequence with e-values lower than e-value of 10^–43^ could be identified within a tblastn analysis with the standard tblastn parameter values, we concluded the presence of the EMCP. Sequences with similarity to Icl of *Escherichia coli* with a boundary e-value of 10^–14^ served as marker genes for the glyoxylate shunt.

If the candidate carried crotonyl-CoA carboxylase/reductase and lacked isocitrate lyase, they were included in the further analysis. Subsequent analysis of the literature was performed to identify the strains capable of C1 and C2 substrate utilization and the strains for which growth on C1 and C2 substrates has already been described in the literature before were selected for further investigations.

### Bacterial strains and media

*M. extorquens* AM1 (DSM1338) and all strains identified in this work were obtained from DSMZ (German Collection of Microorganisms and Cell Cultures, Braunschweig, Germany) and propagated in the media recommended by DSMZ or previously described in literature for every specific strain. *Xanthobacter autotrophicus* DSM1618 and *X. autotrophicus* DSM432 were cultivated in medium 1, *Hyphomicrobium* sp. DSM3646 in medium 162, *Ruegeria pomeroi* DSM15171 in medium 974 (Salgado et al. 2014) and *Pseudonocardia dioxanivorans* DSM44775 in medium 553, respectively. Cultivation of *Lactobacillus reuteri* DSM20016 was performed in medium 11.

### Media and cultivation conditions for vitamin B_12_ production

Mineral medium used for the analysis of cobalamin production with the investigated strains was previously described by (Peyraud et al. 2009). Briefly, mineral salts contained 1.62 g/L NH_4_Cl and 0.2 g/L MgSO_4_, buffer was composed of 2.21 g/L K_2_HPO_4_ and 1.25 g/L NaH_2_PO_4_ × 2H_2_O (pH 7.1), and the following trace elements were used: 15 mg/L Na_2_EDTAx2H_2_O, 4.5 mg/L ZnSO_4_ × 7H_2_O, 0.3 mg/L CoCl_2_ × 6H_2_O, 1 mg/L MnCl_2_ × 4H_2_O, 1 mg/L H_3_BO_3_, 0.4 mg/L Na_2_MoO_4_ × 2H_2_O, 3 mg/L FeSO_4_ × 7H_2_O and 0.3 mg/L CuSO_4_ × 5H_2_; CaCl_2_ was used in the final concentration of 2.5 mg/L. The standard medium was supplemented with 0.5% (v/v) ethanol for *R. pomeroyi* DSM15171 and 0.5% (v/v) methanol for all other strains as the sole source of carbon and energy, 1.5% (w/v) agar was added if solid medium was used. In the medium optimization experiments with *Hyphomicrobium* sp. DSM3646 the amount of methanol, CaCl2 and of each component of mineral salts, buffer and trace elements was reduced or increased twice.

The strains were cultivated on agar plates or in 300 mL shaking flasks for 6 days at 30 °C under aeration and shaking at 180 rpm.

### Vitamin B_12_ extraction and purification

For the cobalamin analysis from the solid media the cells obtained after the incubation were scratched with the spatula from the agar surface and the whole biomass was used for cobalamin extraction. For the cobalamin analysis from the liquid cultures, 25 mL of the broth was harvested by centrifugation at 3.150 × g for 30 min. The cobalamin was extracted in the cyano-form and the cell pellets were resuspended in 10 mL of acetate buffer (4.1 g/L of sodium acetate, pH adjusted to 4.5 with acetic acid) containing 100 µL of 1% KCN. After the incubation in a water bath at 98 °C for 30 min the samples were cooled on ice for 30 min and centrifuged again. Vitamin B_12_ was purified from the obtained supernatants using BAKERBOND spe™ C18 columns JB7020-03 (J. T. Baker, VWR, Germany) according to the manufacturer’s instructions. The extracts were then syringe filtered (0.2 µM), dried at 60 °C under vacuum and resuspended in 100 µL of deionized H_2_O.

### Analysis of vitamin B_12_ via liquid chromatography-mass spectrometry (LC–MS/MS)

Analysis of the samples was performed with a triple quadrupole LCMS-8045 (Shimadzu, Germany) and the Lab Solutions Analysis Software (Shimadzu, Germany) was used for data acquisition and analysis. A Luna® Omega 3 µm PS C18 100 Å Column (Phenomenex, Germany) was operated with 0.1% formic acid in water (solvent A) and 0.1% formic acid in acetonitrile (solvent B). All solvents used were of LC/MS grade and (Carl Roth, Germany). The following LC time program was maintained during the method run: 0–3 min 18–32% B, 3–3.1 min 32–95% B, 3.1–4.1 min 95% B, 4.1–4.3 min 95–18% B, 4.3–7 min 18% B. The flow rate was 0.4 mL/min and the column temperature was maintained at 40 °C. The MS analysis was carried out in positive ion mode using electrospray ionization (ESI) under following parameters: nebulizing gas flow 3 L/min, drying gas flow 10 L/min, interface temperature 300 °C, desolvation line temperature 250 °C and heat block temperature 400 °C. The mass spectrometer was run in multiple reaction monitoring (MRM) mode for cobalamin (MRM ( +) m/z 678.40 → m/z 146.95, m/z 678.40 → m/z 359.10) and pseudocobalamin (MRM ( +) m/z 672.75 → m/z 136.05, m/z 672.75 → m/z 348.05) with method parameters (collision energies, dwell times and exact m/z values) optimized with the software for each transition. The injection volume was 1 µL and the quantification of vitamin B_12_ in the samples was performed using a calibration curve obtained from a set of cyanocobalamin standards (Merck, Germany). Since pseudovitamin B_12_ is commercially not available as analytical standard, the cell extract of *L. reuteri* containing pseudovitamin (Santos et al. 2007) was used as reference material.

### Cultivation of *Hyphomicrobium sp.* DSM3646 in BioLector microbioreactor

The pre-cultures of *Hyphomicrobium* sp. DSM3646 were grown in MeOH minimal medium for 48 h and used for the inoculation of 1 mL medium at the starting OD_600_ of 0.1. The cultivation was carried out in a BioLector® MB system (m2p-labs, Germany) in MTP-48 FlowerPlates® with pH optodes at 30 °C, 1000 rpm and 95% humidity. The growth of the cultures was monitored online by scattered light signal measurement.

### Statistical analysis

If not stated otherwise, all experiments were repeated in biological triplicates. Data are presented as the mean value ± standard deviation. Two-sample t-test was used to determine the significance of the difference between the means. Values were considered significant at *p* < 0.05. The analyses were performed using OriginPro® data analysis software.

## Results

### Confirmation of cobalamin production with *M. extorquens* AM1 by the means of LC–MS/MS

**Fig. 1:**
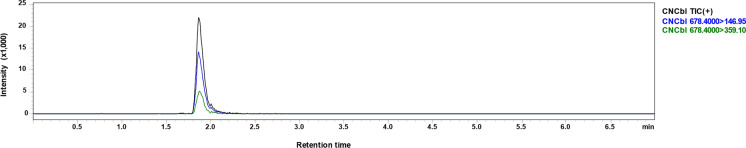
Detection of vitamin B_12_ in the cell extract of *M. extorquens* AM1. Shown is an MRM-chromatogram of the cell extract obtained from cultivation of *M. extorquens* AM1 in minimal medium with 0.5% (v/v) methanol

Firstly, we aimed to validate our hypothesis about potentially high vitamin B_12_ levels in microorganisms with an essential function of cobalamin-dependent mutases in the EMCP as primary metabolism pathway. Therefore, we confirmed cobalamin production in the cells of *M. extorquens* AM1 upon growth with methanol as sole carbon source. The performed LC–MS/MS analysis of the obtained cell extracts demonstrated characteristic masses of cyanocobalamin (Fig. 1).

The detected parent ion signal with m/z of 678.40 and fragment ion signals with m/z 146.95 and m/z 359.10, which correspond to [DMBI + H]^+^ and [DMBI + sugar + PO_3_ + H]^+^, respectively, provided evidence for vitamin B_12_ synthesis in *M. extorquens* AM1.

### Identification of the candidate strains

To identify other microorganisms with a high vitamin B_12_ demand and potential for high vitamin B_12_ synthesis, we aimed to find bacterial strains with essential function of the cobalamin-dependent mutases in the EMCP. Among all at the time of the search available bacterial genomes, 65 strains could be identified, which contained a Ccr-encoding gene and lacked an Icl-encoding gene according to the presence of sequences with similarity to the used queries (Supplementary Table S1). Analysis of literature data revealed that *X. autotrophicus* DSM1618 and *X. autotrophicus* DSM432, *Hyphomicrobium* sp. DSM3646 and *P. dioxanivorans* DSM44775 should be able to grow on methanol as sole carbon source (Ginkel and Bont 1986; Harder et al. 1973; Grostern and Alvarez-Cohen 2013), while *R. pomeroyi* DSM15171 was reported to be capable of ethanol utilization (González et al. 2003). As the EMCP should be only essential during growth with C1 or C2 carbon sources, we focused on these five bacteria for further investigations.

### Verification of active vitamin B_12_ production in the cells of selected strains

For the analysis of vitamin B_12_ production the five selected strains were cultivated on agar plates with minimal medium containing methanol or ethanol as the sole carbon source. In contrast to previously reported data (González et al. 2003), we could not detect growth of *R. pomeroyi* DSM15171 in the respective medium. *X. autotrophicus* DSM1618, *X. autotrophicus* DSM432, *Hyphomicrobium* sp. DSM3646 and *P. dioxanivorans* DSM44775 were able to grow on agar plates with solid minimal medium containing 0.5% (v/v) methanol and were analyzed for the ability to produce the active form of vitamin B_12_ and pseudocobalamin.

**Fig. 2:**
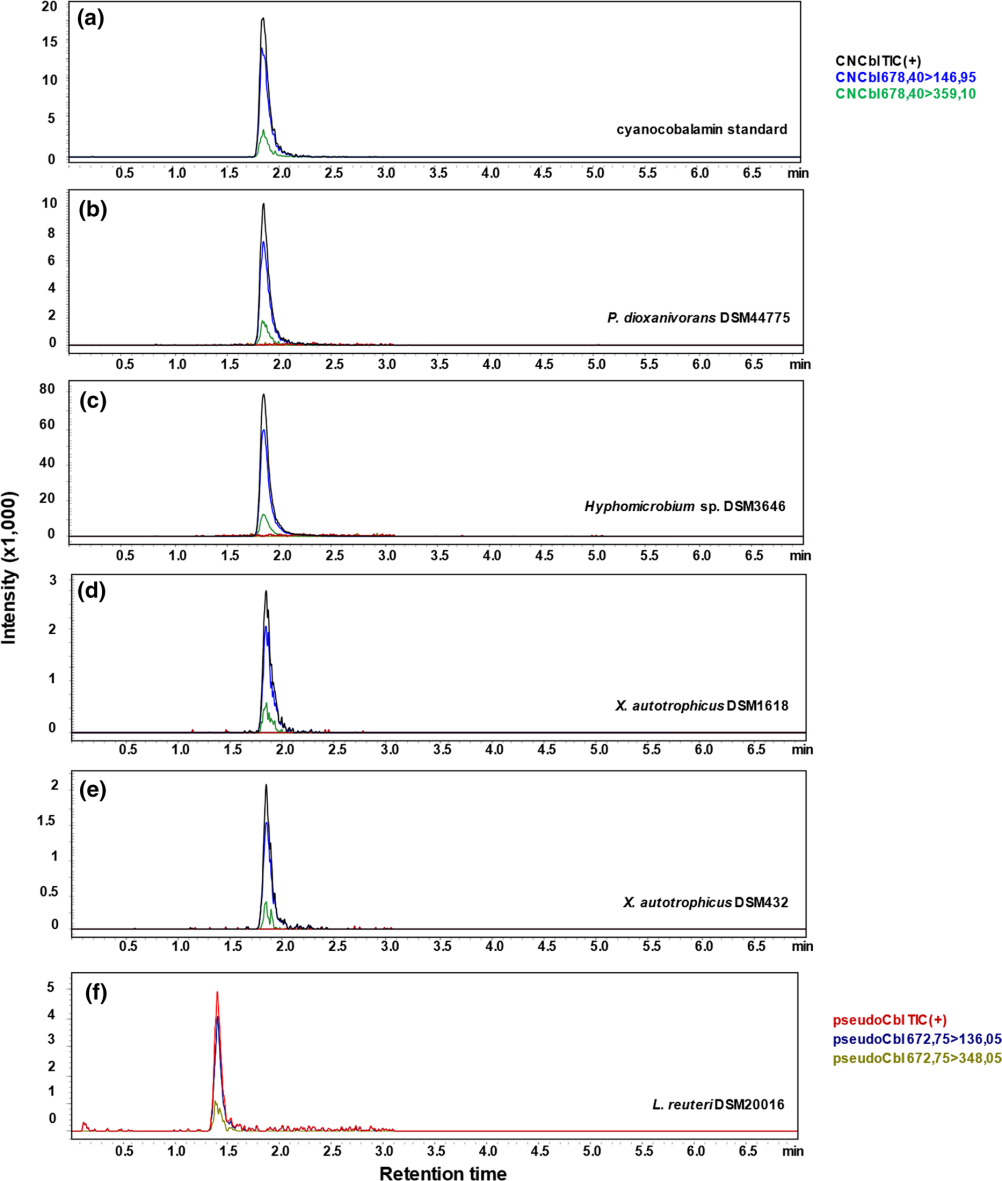
LCMS/MS analysis of cyanocobalamin, pseudocobalamin B_12_ in the obtained extracts. Shown are MRM chromatograms of **a** the cyanocobalamin standard, **b–e** extracts of *P. dioxanivorans* DSM44775, *Hyphomicrobium* sp. DSM3646, *X. autotrophicus* DSM1618 and *X. autotrophicus* DSM432 cultivated on the solid minimal medium, **f** pseudocobalamin detected in the extract of *L.reuteri* DSM20016

As Fig. 2 shows, due to the differences in their ligand structure, the chromatograms of the active cobalamin and pseudovitamin B_12_ demonstrate characteristic masses of parent and fragment ions and also differences in their retention times. Analysis of the cell extracts obtained after the cultivation in the minimal methanol medium demonstrated a peak at 1.77 min showing the parent ion signal with m/z of 678.40 and fragment ion signals with m/z 146.95 and m/z 359.10 present in the chromatograms of the samples from all strains. These values correspond to the retention time and fragmentation pattern of cyanocobalamin and confirm the ability of the identified strains for the synthesis of active vitamin B_12_.

Moreover, active cobalamin was the only form produced by all strains. The presence of pseudovitamin B_12_ could be excluded, since its characteristic peak at 1.38 min showing the parent ion signal with m/z of 672.75 and fragment ion signals with m/z 136.05 and m/z 348.05 was not present in the chromatograms of the four strains.

### Comparison of vitamin B_12_ production by the selected strains

**Fig. 3:**
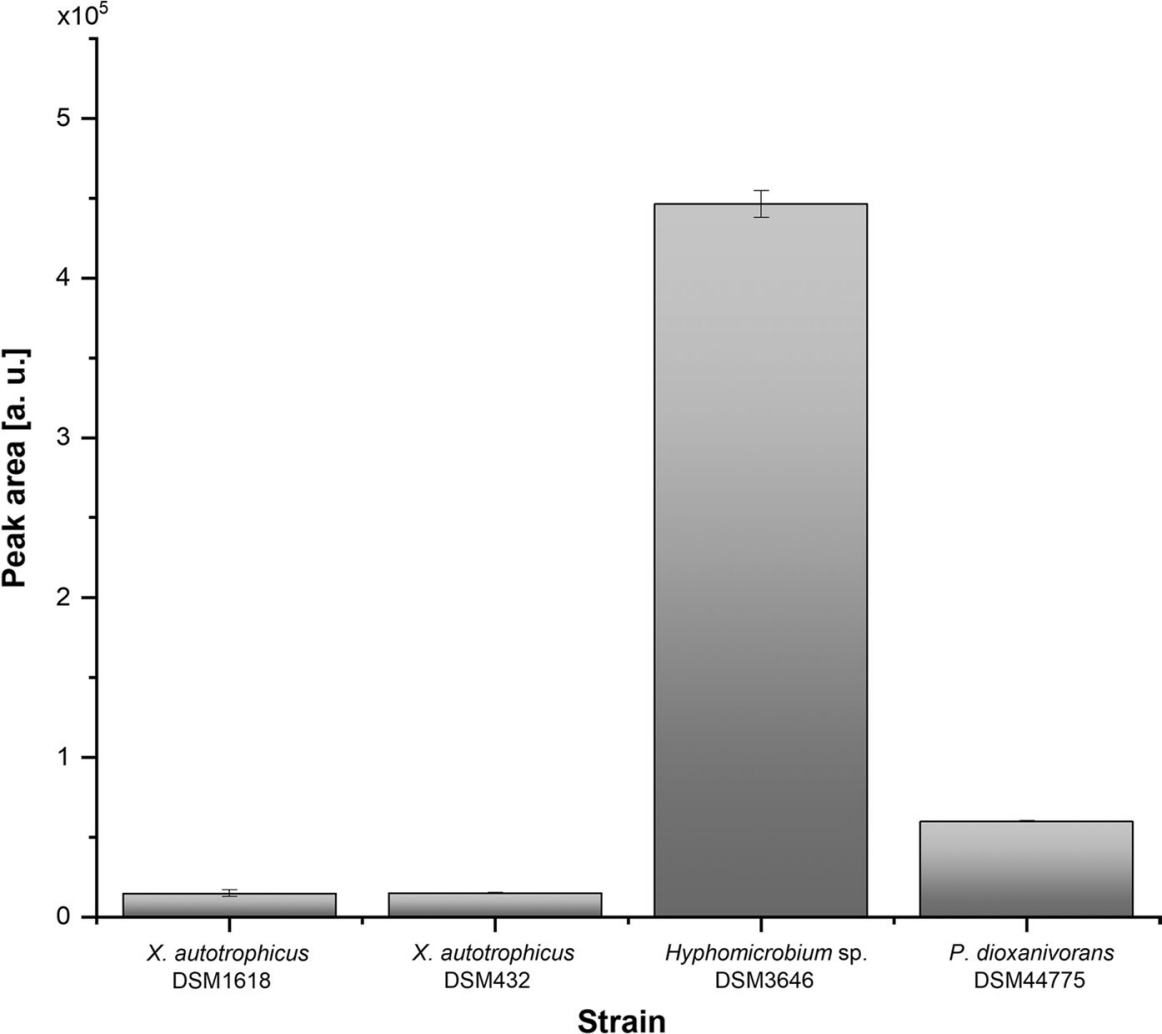
Comparison of the total ion chromatogram (TIC) peak areas of the cyanocobalamin produced by the selected strains. Data are shown as mean values of technical replicates ± standard deviation (n = 3)

In order to identify strains with high vitamin B_12_ production capabilities, the cyanocobalamin peak areas obtained from the cell extracts were compared. As Fig. 3 shows, the peak areas varied greatly between the samples from the four strains. Although all bacteria were capable of vitamin B_12_ production, the peak area corresponding to the cell extract of *Hyphomicrobium* sp. DSM3646 was 7.5 times higher than the peak area of *P. dioxanivorans* DSM44775 cell extract and nearly 30 times higher than those determined for the cell extracts of *X. autotrophicus* DSM1618 and *X. autotrophicus* DSM432.

In addition to the highest vitamin B_12_ peak areas, only *Hyphomicrobium* sp. DSM3646 was able to grow in liquid medium, while no growth in liquid medium was observed for *P. dioxanivorans* DSM44775, *X. autotrophicus* DSM1618 and *X. autotrophicus* DSM432.

### Vitamin B_12_ production by ***Hyphomicrobium*** sp. DSM3646 and ***M. extorquens*** AM1

**Fig. 4:**
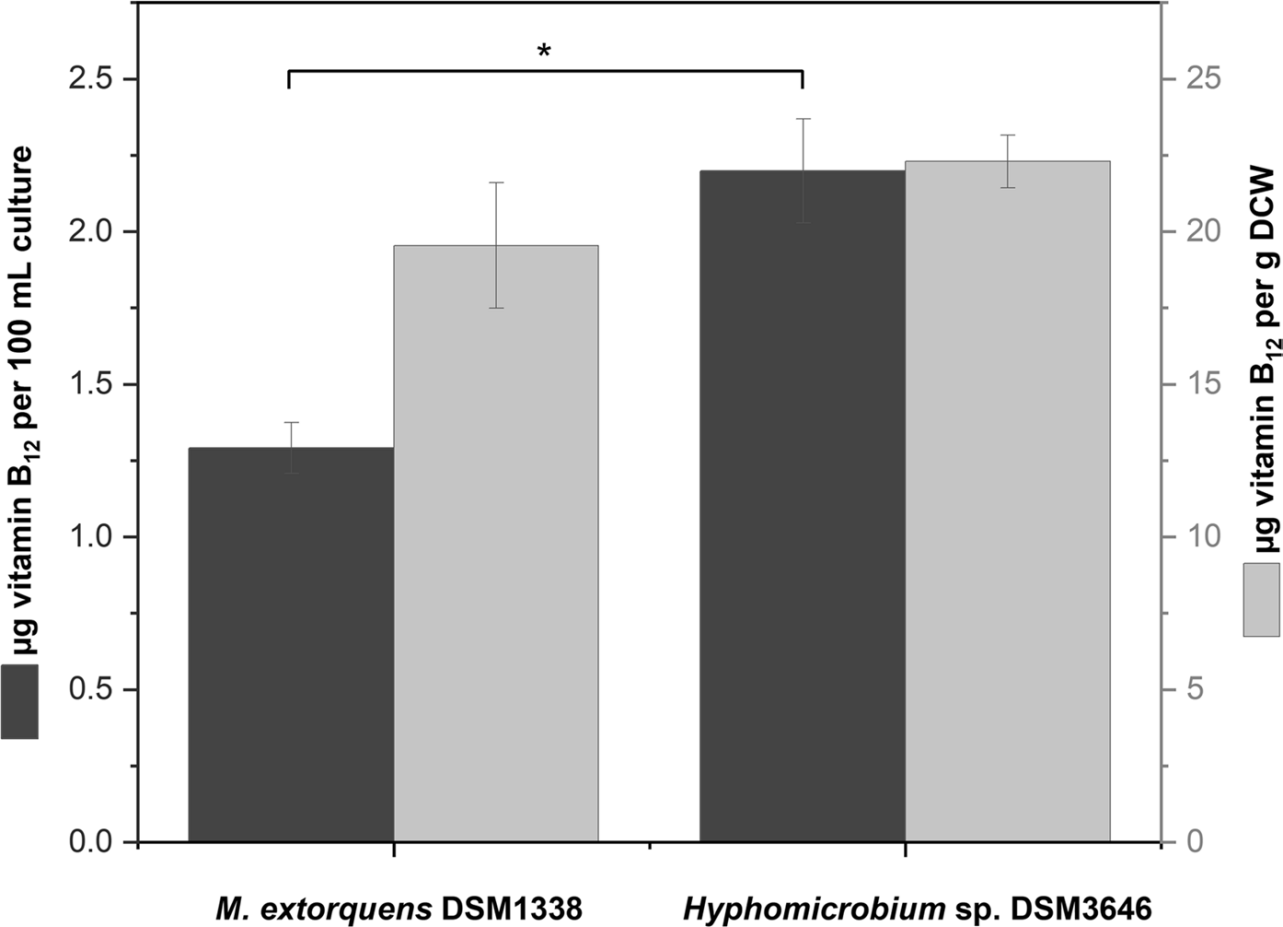
Comparison of vitamin B_12_ amounts produced with *Hyphomicrobium* sp. DSM3646 and *M. extorquens* AM1 in the minimal medium with 0.5% methanol. The data are represented as the mean values and standard deviations of three biological replicates. Two-sample t-test was used for statistical comparison between the strains, differences considered as significant at *p* < 0.05 are denoted by an asterisk

As a subsequent step, we decided to compare the initial *M. extorquens* AM1 strain with the newly identified *Hyphomicrobium* sp. DSM3646 for their ability to synthesize vitamin B_12_ in the minimal methanol medium (Fig. 4).

The OD_600_ value of 2.97 ± 0.06 measured in the culture of *Hyphomicrobium* sp. DSM3646 was significantly higher than 2.06 ± 0.06 reached by *M. extorquens* AM1. Therefore, the amount of cobalamin was expressed per g dry cell weight (DCW) for the relative comparison. The value detected in the culture of *Hyphomicrobium* sp. DSM3646 was slightly higher than the amounts reached by *M. extorquens* AM1, nevertheless, these differences were not significant.

However, the subsequently performed t-test for the cobalamin concentrations per 100 mL culture showed that the vitamin B_12_ amounts varied significantly between the producing strains. The yield of vitamin B_12_ achieved by *Hyphomicrobium* sp. DSM3646 was 2.2 ± 0.17 µg per 100 mL culture, which is 1.7 times higher than 1.29 ± 0.08 µg per 100 mL culture produced by *M. extorquens* AM1. Due to the higher volume yield of vitamin B_12_, which is probably caused by higher biomass formation, *Hyphomicrobium* sp. DSM3646 was chosen for further investigation.

### Optimization of *Hyphomicrobium* sp. DSM3646 cultivation medium

The components of the minimal medium were evaluated to identify the factors that have effect on biomass accumulation with *Hyphomicrobium* sp. DSM3646. For this purpose, we altered the concentration of methanol, buffer, CaCl_2_, trace elements and mineral salts in the standard medium and tested media in which the amount of each single component was increased or reduced twice. The standard minimal medium was used as reference and the growth behavior of *Hyphomicrobium* sp. DSM3646 under the standard and altered conditions was investigated.

**Fig. 5:**
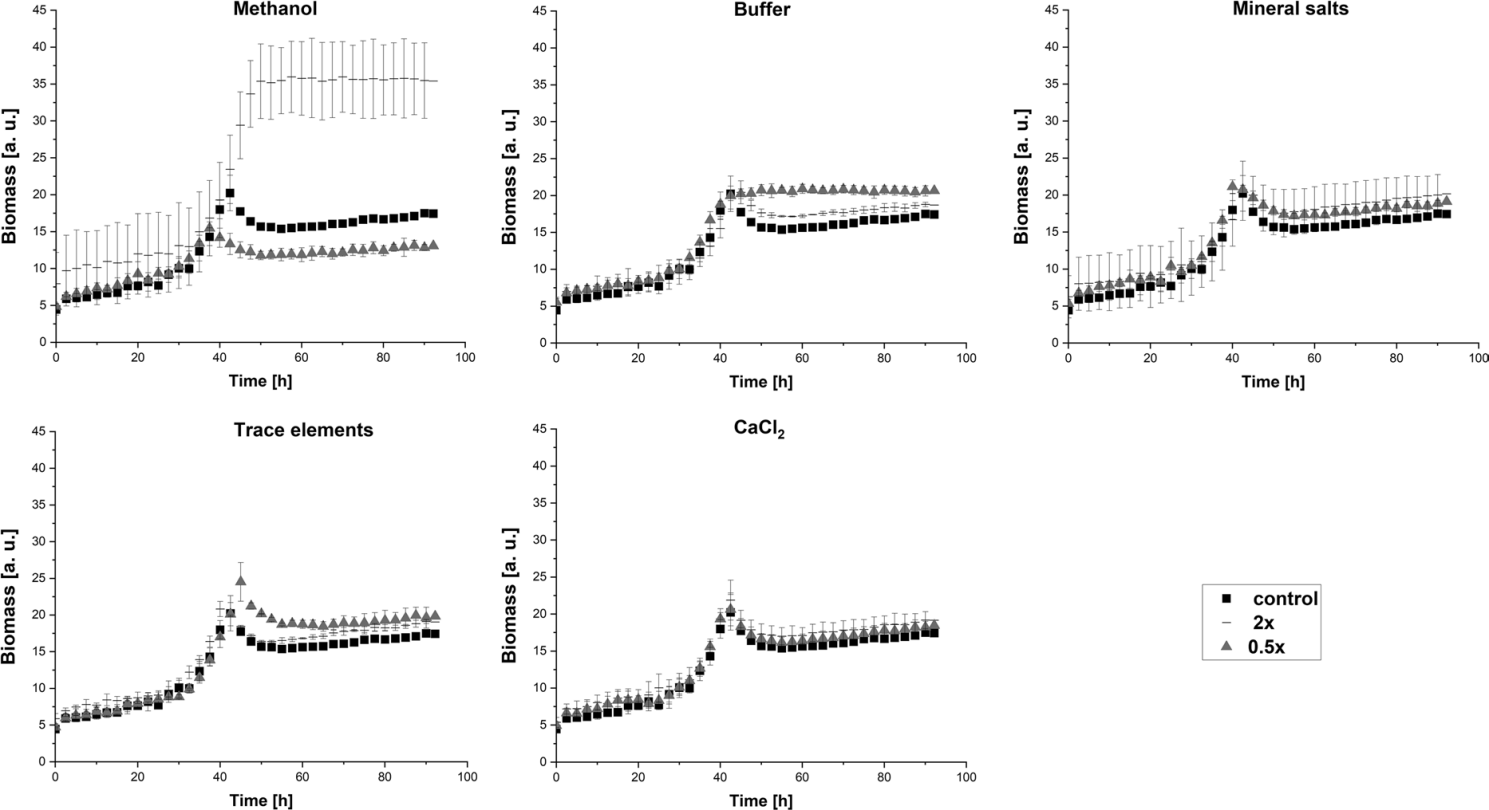
Influence of the methanol mineral medium components on the growth of *Hyphomicrobium* sp. DSM3646. The cultivations were performed in BioLector microbioreactor, the data points represent the mean values and standard deviations of three biological replicates

The effect of different media components on the growth of the organism is presented in Fig. 5. Among all investigated components, methanol was identified as the factor having the greatest influence on the growth of *Hyphomicrobium* sp. DSM3646. The results show that both increase and decrease of methanol concentration, had a significant effect on biomass formation. The values observed in the medium with 1% methanol reached above 35 a. u. after 50 h, while 0.25% of methanol in the medium resulted in a scattered light signal of 12 a. u., which was significantly lower than the values achieved in the standard medium with 0.5% methanol. As Fig. 5 shows, the changes in CaCl_2_ concentration had no effect on the growth behavior of *Hyphomicrobium* sp. DSM3646, while only slight biomass increase was observed when buffer, trace elements and mineral salts concentration was increased.

**Fig. 6:**
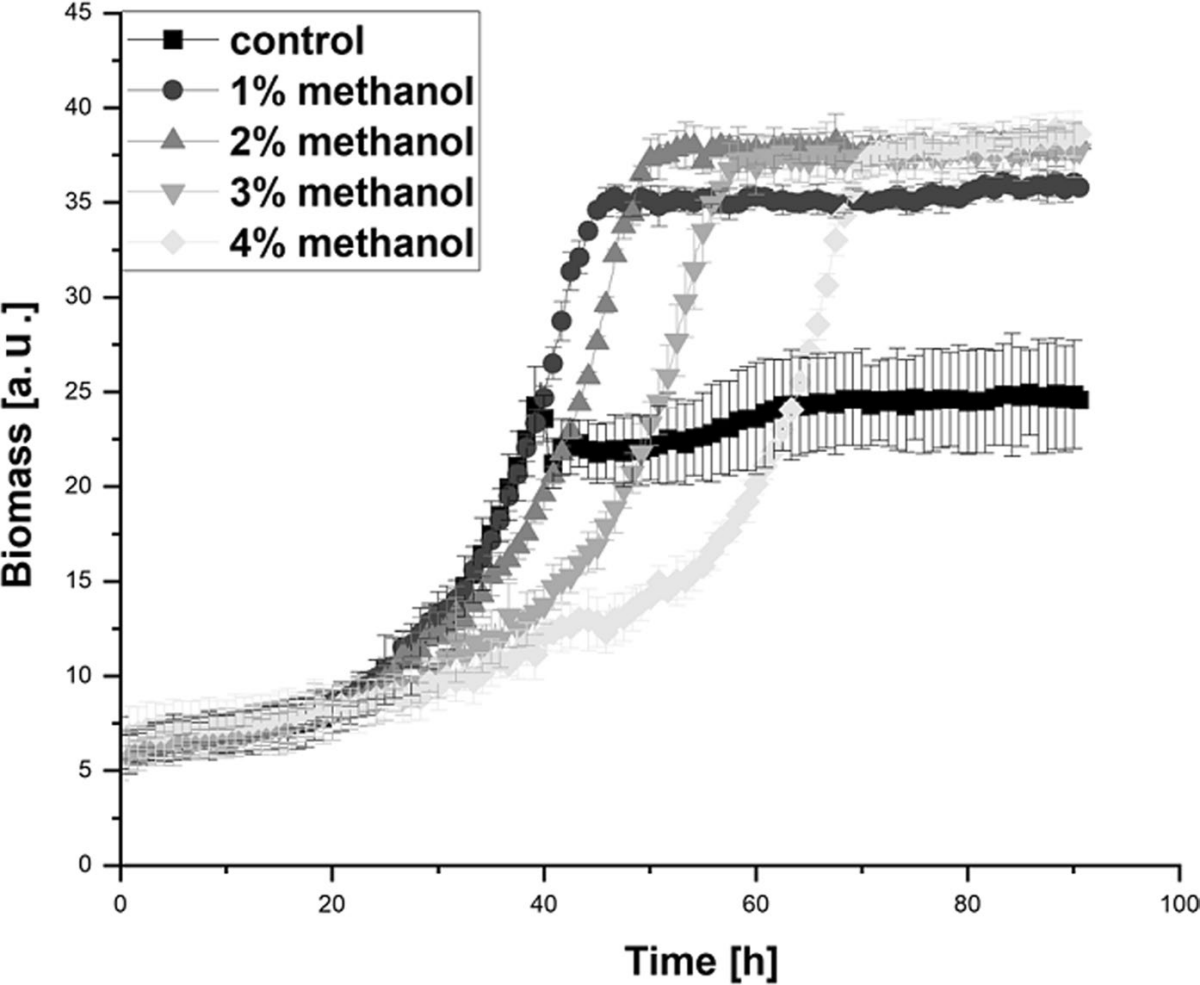
Growth curves of *Hyphomicrobium* sp. DSM3646 with increased methanol concentrations of 1–4%, standard medium with 0.5% methanol used as control. The cultivations were performed in BioLector microbioreactor, the data points represent the mean values and standard deviations of three biological replicates

For the determination of the optimal methanol concentration, the growth of *Hyphomicrobium* sp. DSM3646 in the standard minimal medium containing 0.5% methanol (control) was compared with the growth in media containing 1%, 2%, 3% or 4% of methanol (Fig. 6). All changes in methanol concentrations affected the growth resulting in a maximal scattered light signal 1.6 times higher in comparison to the reference medium. The highest scattered light signal was determined in the media with methanol concentrations of 2% and higher, while no further signal increase was detected in the media containing 3% and 4% of methanol.

Despite the positive influence on biomass formation, higher methanol concentration led to elongated lag phases. *Hyphomicrobium* sp. DSM3646 could reach the maximum scattered light signal after 40 h in the control medium, while it took 45 h, 50 h, 58 h and 70 h in the media with 1%, 2%, 3% and 4% of methanol, respectively. Overall, most efficient biomass formation with only slight growth inhibition was observed in the medium with 2% of methanol. Therefore, it was chosen as starting point for the following experiment with *Hyphomicrobium* sp. DSM3646.

**Fig. 7:**
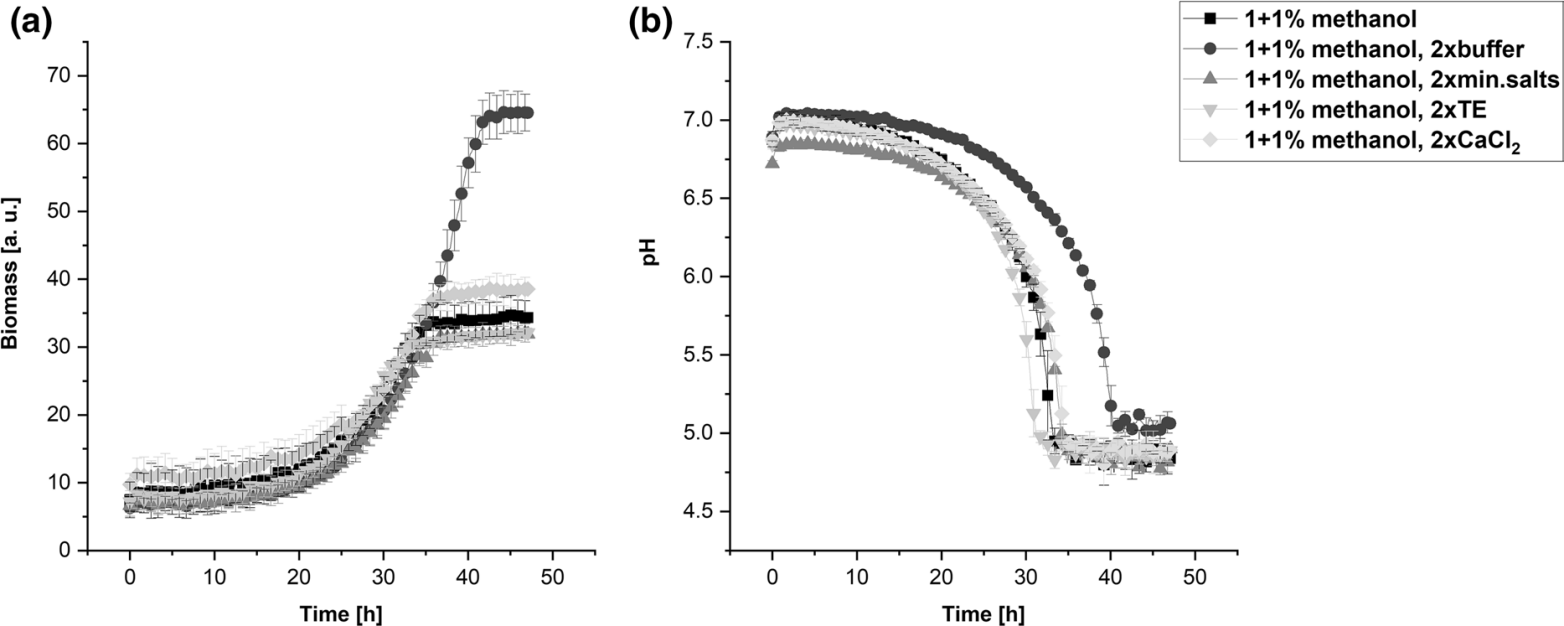
Growth curves **a** of *Hyphomicrobium* sp. DSM3646 and the corresponding pH changes **b** in the medium with 2% methanol combined with the two-fold increased concentration of the buffer, mineral salts, trace elements and CaCl_2_. The cultivations were performed in BioLector microbioreactor, the data points represent the mean values and standard deviations of three biological replicates

Since we aimed to avoid the inhibitory effect of methanol in this medium, we decided to inoculate the cells in the medium containing 1% of methanol and add further 1% of methanol in the mid-exponential phase of growth. These growth conditions were then investigated in combination with the doubled amounts of each other medium component (Fig. 7a).

Stepwise methanol addition enabled to achieve the maximum scattered light signal after 35 h, which was comparable with the medium with standard methanol concentration (Fig. 6). Among all tested components, the increased amount of the buffer had the greatest influence on the biomass formation leading to the significant increase of the scattered light signal over 64 a. u. This value was 1.7 times higher than in the medium with the standard buffer concentration and 2% methanol and 2.8 times higher than the signal reached in the medium with standard buffer concentration and 0.5% methanol. We proposed that pH was the growth-limiting factor in the media with the standard buffer concentration and performed pH measurement in all tested media. As. Figure 7b shows, the pH value changes correlated with the growth behavior of the cultures. The pH drop observed in the media with the standard buffer amount led to a pH value of 5 after 35 h, which was the time point when the stationary growth phase was reached. In case of the medium with the increased buffer concentration, the same trend was observed 5 h later, when acidic pH and the stationary growth phase were achieved after 40 h of cultivation.

As no significant effect on the growth of the cells was observed for concentration changes of other medium components, the medium with increased buffer amount supplemented with 2% of methanol was taken for the further investigation of vitamin B_12_ production.

### Production of vitamin B_12_ by ***Hyphomicrobium*** sp. DSM3646 in the standard and optimized minimal medium

**Fig. 8:**
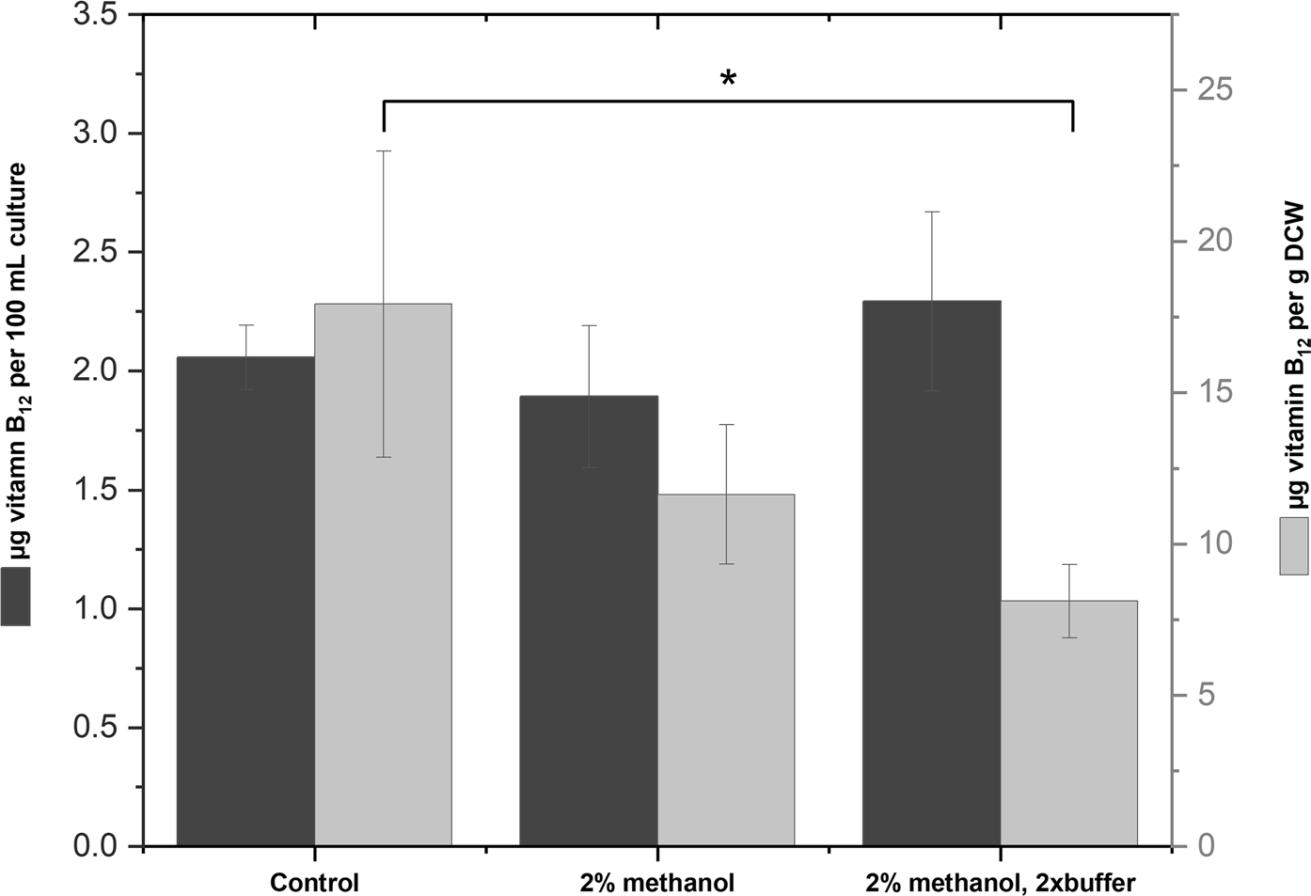
Comparison of vitamin B_12_ production in the standard medium (control), in the medium with 2% methanol and in the medium with 2% methanol and twofold increased buffer concentration. The data are shown as the mean values and standard deviations of three biological replicates. Two-sample t-test was used for statistical comparison between the media, differences considered as significant at *p* < 0.05 are denoted by an asterisk

The levels of produced active vitamin B_12_ were determined for cells in the late stationary phase of growth and the results were compared for the standard medium and for the media with increased methanol and buffer concentrations (Fig. 8).

Although the optimization of the cultivation medium significantly influenced the biomass formation, this did not increase the yield of vitamin B_12_ compared to the standard medium. The amounts of cobalamin in 2% methanol medium were slightly lower than those reached in the standard medium, while minor increase of cobalamin content was detected in the 2% methanol medium with the doubled buffer concentration. However, the unpaired t-test revealed that the differences between the values detected in the tested media were not significant.

Since the OD values varied greatly between 3.2 ± 0.17, 6.5 ± 0.2 and 9.93 ± 0.76 for the standard, 2% methanol and 2% methanol medium with the increased buffer, respectively, we calculated the amount of cobalamin per g DCW for the purpose of relative comparison. The highest yield of 17.9 ± 5.05 µg per g DCW was obtained in the standard medium, which was significantly higher than 8.11 ± 1.21 µg per g DCW of vitamin B_12_ achieved in the 2% methanol medium with the increased buffer concentration.

## Discussion

Although this issue has not been investigated so far to our knowlewdge, one might assume, that organisms with high vitamin B_12_ synthesis capability have an essential need for the cofactor itself. The reduction of pyruvate to propionate, a key reaction of *P. freudenreichii* metabolism, occurs in the Wood–Werkman cycle, which includes coenzyme B_12_-dependent methylmalonyl-CoA mutase (Thierry et al. 2011). The vitamin B_12_-producing plant symbiont *Sinorhizobium meliloti* (Burton and Lochhead 1952) requires a cobalamin-dependent ribonucleotide reductase to establish the symbiosis with its plant host (Taga and Walker 2011). Similar to these microorganisms, methylotrophs with cobalamin-dependent enzymes in the EMCP seemed to be promising as a new source for vitamin B_12_. Our findings show that investigation of microorganisms with essential functions of cobalamin in their primary metabolism is a successful strategy for the identification of new vitamin B_12_ producers.

Although few preliminary reports have already described production of vitamin B_12_ in methylotrophic bacteria (Ivanova et al. 2006; Danilova et al. 2004), the total content of corrinoids was measured with *Escherichia coli* strain 113–3 auxotrophic for vitamin B_12_ in those studies. However, this microbiological assay was shown to respond not only to vitamin B_12_ but also to its analogues (Ford 1959), which is why a comparison of these previously reported data with the results obtained for the active vitamin B_12_ in this study was not performed. Therefore, if the goal is to produce pure cobalamin or fortify food for human nutrition purposes, it is important to determine the type of the cobalamin synthesized by the microorganisms (Chamlagain et al. 2017). In our study, we report on the identification of the active vitamin B_12_ with a sensitive LC–MS/MS method in the cell extracts of *M. extorquens* AM1. Moreover, based on these data, we were also able to identify other strains with high vitamin B_12_ production capability, which have not been described in literature before. Our analyses show, that the active B_12_ is the form exclusively produced by all identified candidates (Fig. 2) with *Hyphomicrobium* sp. DSM3646 showing the highest content under the conditions tested (Fig. 3). Direct comparison of vitamin B_12_ production with *M. extorquens* AM1 and the newly identified *Hyphomicrobium* sp. DSM3646 demonstrated that the latter is the most suitable strain for vitamin B_12_ production.

We performed *Hyphomicrobium* sp. DSM3646 growth experiments which identified conditions enabling higher biomass formation compared to the standard medium. Although increased methanol concentration positively influenced biomass formation, the analysis of vitamin B_12_ synthesis revealed that the highest cobalamin amounts were produced in the standard medium. This implies that vitamin B_12_ production does not directly correlate with biomass formation and makes the standard medium more advantageous for vitamin B_12_ production. Moreover, we proposed that acidification can be another factor limiting growth of *Hyphomicrobium* sp. DSM3646. To examine this issue, we measured pH and detected significant differences in values between the 2% methanol medium with standard or increased buffer concentrations (Fig. 7b). Nevertheless, high biomass accumulation observed in the latter medium was accompanied with production of lower vitamin B_12_ amounts. This observation underlined the finding that the standard medium is the most appropriate for vitamin B_12_ production. Cobalt can also be a limiting factor for vitamin B_12_ synthesis, since it is necessary for the corrinoid ring formation, which is why it is often supplied during vitamin B_12_ production (Chamlagain et al. 2016; Deptula et al. 2015). Since cobalt was added into the medium composition in this study and the used concentrations exceed greatly the amounts of the obtained vitamin B_12_, we propose that there should be another factor limiting vitamin B_12_ production in *Hyphomicrobium* sp. DSM3646.

The achieved cobalamin levels are lower than the concentration range of 45–335 µg per 100 mL culture reported previously for various *P. freudenreichii* strains (Chamlagain et al. 2016; Deptula et al. 2017). However, it is worth mentioning, that the values described for *P. freudenreichii* were obtained after optimization of the production process, while the current study describes the first attempts on vitamin B_12_ production with *Hyphomicrobium* sp. DSM3646. Moreover, the completely aerobic cultivation process for vitamin B_12_ production with *Hyphomicrobium* sp. DSM3646 is advantageous in comparison to the two-step cultivation procedure necessary for vitamin B_12_ synthesis with *P. freudenreichii* (Chamlagain et al. 2016, 2017; Deptula et al. 2017)*.* Since production of active vitamin B_12_ with *Hyphomicrobium* sp. DSM3646 was demonstrated in this work, it makes it attractive for possible enrichment of plant-based foods with vitamin B_12_. For this reason, further investigations on *Hyphomicrobium* sp. DSM3646 safety should be made for its possible use in food fortification. Finally, the use of low-cost methanol mineral medium for vitamin B_12_ production with *Hyphomicrobium* sp. DSM3646 can contribute to the development of an economically viable process. The economic benefits of such process can be evaluated in future studies after optimization of its productivity against the commonly used strains.

## Supplementary Information

Below is the link to the electronic supplementary material.

Supplementary file1 (DOCX 19 KB)

## Data Availability

The data generated during the current study are available from the corresponding author on reasonable request.
