# Supplementary material for: Methylotrophic bacteria with cobalamin-dependent mutases in primary metabolism as potential strains for vitamin B12 production
Source: Antonie Van Leeuwenhoek. 2022 Nov 17;116(3):207–20. doi: 10.1007/s10482-022-01795-9 (PMC9925536; doi:10.1007/s10482-022-01795-9)
Supplement: Supplementary file 1 — Supplementary file1 (DOCX 19 KB) [file 10482_2022_1795_MOESM1_ESM.docx]

| **Accession Number** | **Organism** |
| --- | --- |
| NC_018485.1 | Methylocystis sp. SC2 |
| NC_009720.1 | Xanthobacter autotrophicus Py2 |
| NC_009937.1 | Azorhizobium caulinodans ORS 571 |
| NC_015717.1 | Hyphomicrobium sp. MC1 |
| NC_007626.1 | Magnetospirillum magneticum AMB-1 |
| NC_015259.1 | Polymorphum gilvum SL003B-26A1 |
| NC_008209.1 | Roseobacter denitrificans OCh |
| NC_009049.1 | Rhodobacter sphaeroides ATCC 17029 |
| NC_015730.1 | Roseobacter litoralis Och 149 |
| NC_007493.1 | Rhodobacter sphaeroides 2.4.1 |
| NC_003911.1 | Ruegeria pomeroyi DSS-3 |
| NC_014664.1 | Rhodomicrobium vannielii ATCC 17100 |
| NC_018290.1 | Phaeobacter gallaeciensis DSM 17395 |
| NC_016642.1 | Pseudovibrio sp. FO-BEG1 |
| NC_018286.1 | Phaeobacter gallaeciensis 2.10 |
| NC_017956.1 | Tistrella mobilis KA081020-065 |
| NC_009952.1 | Dinoroseobacter shibae DFL 12 |
| NC_010338.1 | Caulobacter sp. K31 |
| NC_007802.1 | Jannaschia sp. CCS1 |
| NC_017584.1 | Rhodospirillum rubrum F11 |
| NC_007643.1 | Rhodospirillum rubrum ATCC 11170 |
| NC_014100.1 | Caulobacter segnis ATCC 21756 |
| NC_017059.1 | Rhodospirillum photometricum DSM 122 |
| NC_012982.1 | \| Hirschia baltica ATCC 49814 |
| NC_008392.1 | Burkholderia ambifaria AMMD |
| NC_011883.1 | Desulfovibrio desulfuricans subsp. desulfuricans |
| NC_019673.1 | Saccharothrix espanaensis DSM 44229 |
| NC_018020.1 | Turneriella parva DSM 21527 |
| NC_014830.1 | Intrasporangium calvum DSM 43043 |
| NC_010602.1 | Leptospira biflexa serovar Patoc strain ‘Patoc I’ |
| NC_010842.1 | Leptospira biflexa serovar Patoc strain ‘Patoc (Ames)’ |
| NC_005823.1 | Leptospira interrogans serovar Copenhageni |
| NC_004342.2 | Leptospira interrogans serovar Lai str. 56601 |
| NC_013757.1 | Geodermatophilus obscurus DSM 43160 |
| NC_008508.1 | Leptospira borgpetersenii serovar Hardjo-bovis str. 550 |
| NC_020291.1 | Clostridium saccharoperbutylacetonicum N1-4 |
| NC_017765.1 | Streptomyces hygroscopicus subsp. jinggangensis 5008 |
| NC_013739.1 | Conexibacter woesei DSM 14684 |
| NC_014166.1 | Arcobacter nitrofigilis DSM 7299 |
| NZ_CM000951 | Streptomyces sviceus ATCC 29083 |
| NC_013929.1 | Streptomyces scabiei 87.22 |
| NC_015957.1 | Streptomyces violaceusniger Tu 4113 |
| NC_016582.1 | Streptomyces bingchenggensis BCW-1 |
| NZ_CM001015 | Streptomyces clavuligerus ATCC 27064 |
| NZ_CM001024 | Aeromicrobium marinum DSM 15272 |
| NC_014391.1 | Micromonospora aurantiaca ATCC 27029 |
| NC_014815.1 | Micromonospora sp. L5 |
| NZ_CM000950 | Streptomyces pristinaespiralis ATCC 25486 |
| NC_015312.1 | Pseudonocardia dioxanivorans CB1190 |
| NC_017586.1 | Streptomyces cattleya NRRL 8057 = DSM 46488 |
| NC_016111.1 | Streptomyces cattleya NRRL 8057 |
| NC_016109.1 | Kitasatospora setae KM-6054 |
| NC_015953.1 | Streptomyces sp. SirexAA-E |
| NC_007777.1 | Frankia sp. CcI3 |
| NC_009921.1 | Frankia sp. EAN1pec |
| NC_016114.1 | Streptomyces flavogriseus ATCC 33331 |
| NC_016943.1 | Blastococcus saxobsidens DD2 |
| NC_013131.1 | Catenulispora acidiphila DSM 44928 |
| NC_015656.1 | Frankia symbiont of Datisca glomerata |
| NC_014666.1 | Frankia sp. EuI1c |
| NC_013124.1 | Acidimicrobium ferrooxidans DSM 10331 |
| NC_008699.1 | Nocardioides sp. JS614 |
| NC_013729.1 | Kribbella flavida DSM 17836 |
| NC_009953.1 | Salinispora arenicola CNS-205 |
| NC_009380.1 | Salinispora tropica CNB-440 |
